# ENgage YOung people earlY (ENYOY): a mixed-method study design for a digital transdiagnostic clinical – and peer- moderated treatment platform for youth with beginning mental health complaints in the Netherlands

**DOI:** 10.1186/s12888-021-03315-x

**Published:** 2021-07-23

**Authors:** M. van Doorn, A. Popma, T. van Amelsvoort, C. McEnery, J. F. Gleeson, F. G. Ory, Jaspers M. W. M., M. Alvarez-Jimenez, D. H. Nieman

**Affiliations:** 1 Department of Psychiatry, Amsterdam University Medical Centers, Amsterdam, The Netherlands; 2 Department of Psychiatry and Neuropsychology Maastricht, Maastricht University, Maastricht, The Netherlands; 3 Centre for Youth Mental Health, The University of Melbourne, Melbourne, Australia; 4 Orygen, Melbourne, Australia; 5 Australian Catholic University, Melbourne, Australia; 6 Buurtzorg Jong, Almelo, The Netherlands

**Keywords:** Indicated prevention, Mental health, E-health, Digital, Youth, Headspace, Stress-biomarkers, Moderated online social therapy (MOST+), Well-being, Early detection and intervention

## Abstract

**Background:**

The onset of mental disorders typically occurs between the ages of 12 and 25, and the burden of mental health problems is the most consequential for this group. Indicated prevention interventions to target individuals with subclinical symptoms to prevent the transition to clinical levels of disorders, even leading to suicide, have shown to be effective. However, the threshold to seek help appears to be high. Digital interventions could offer a solution, especially during the Covid-19 pandemic. This implementation study will investigate the digital indicated prevention intervention ENgage YOung people Early (ENYOY), the Dutch version of the original Moderated Online Social Therapy Platform (MOST+) from Australia. In addition, the relationship between stress biomarkers, symptoms and outcome measures of youth using the platform will be investigated in this study.

**Methods:**

The MOST+ platform will be adapted, translated and developed for the situation in the Netherlands in collaboration with a Youth Panel. A prospective cohort of 125 young people (16–25 years) with beginning mental health complaints will be on the platform and followed for a year, of which 10 participants will have an additional smart watch and 10 participants will be asked to provide feedback about the platform. Data will be collected at baseline and after 3, 6 and 12 months. Outcome measures are Psychological Distress assessed with the Kessler Psychological Distress Scale (K10), Social and occupational functioning (measures by the SOFAS), positive mental health indicators measured by the Positive Health Instrument, stress biomarkers with a smart-watch, website journeys of visitors, and feedback of youth about the platform. It will be a mixed-method study design, containing qualitative and quantitative measures.

**Discussion:**

This trial will specifically address young people with emerging mental health complaints, and offers a new approach for treatment in the Netherlands. Considering the waiting lists in (child and adolescent)-psychiatry and the increase in suicides among youth, early low-threshold and non-stigmatizing help to support young people with emerging psychiatric symptoms is of crucial importance. Moreover, this project aims to bridge the gap between child and adolescent and adult psychiatry.

**Trial registration:**

Netherlands Trial Register ID NL8966, retrospectively registered on the 19th of October 2020.

## Administrative information

**Table:** 

Title	ENgage YOung people earlY (ENYOY): A mixed-method study design for a digital transdiagnostic clinical – and peer- moderated treatment platform for youth with beginning mental health complaints in the Netherlands.
Trial registration	Netherlands Trial Register number ID NL8966 (retrospectively registered 19/10/2020).
Protocol version	Protocol version 1.0, 30 September 2020
Funding	Financial funding by The Netherlands Organisation for Health Research and Development, for personnel, materials, and implementation. File number 60–63600–98-319
Author details	van Doorn M 1, Popma A 1, van Amelsvoort T 2, McEnery C 34, Gleeson JF 45, Öry FG 6, Jaspers MWM 1, Alvarez-Jimenez M 34^,^ Nieman DH 1 1. Amsterdam UMC, Amsterdam, the Netherlands 2. Maastricht University, Department of Psychiatry and Neuropsychology Maastricht, the Netherlands 3. Centre for Youth Mental Health, The University of Melbourne, Australia 4. Orygen, Parkville, Victoria, Australia 5. Healthy Brain and Mind Research Centre, School of Behavioural and Health Sciences, Australian Catholic University, Melbourne, Australia 6. Buurtzorg Jong, Almelo, the Netherlands
Name and contact information for the trial sponsor	The Netherlands Organisation for Health Research and Development **Location** Laan van Nieuw Oost-Indië 334 2593 CE Den Haag, the Netherlands **Postal address** Postbus 93,245 2509 AE Den Haag, the Netherlands Phone 070349 51 11 Fax 070349 51 00 E-mail info@zonmw.nl Administered at the Kamer van Koophandel Den Haag (27365263). The Netherlands Organisation for Health Research and Development is noted as Algemeen Nut Beogende Instelling (ANBI). Fiscal number: 0028.76.528.
Role of sponsor	Monitoring function, check on progress of the study. The sponsor/investigator will submit a summary of the progress of the trial to the accredited METC once a year. Information will be provided on the date of inclusion of the first subject, numbers of subjects included and numbers of subjects that have completed the trial, serious adverse events/serious adverse reactions, other problems, and amendments.

## Introduction

### Background and rationale

About 1 in 4 individuals living in the western world suffer from a mental disorder in a given year [1], and more than half will meet the criteria for a mental disorder at some point in their life [2, 3]. Mental disorders account for a larger proportion of disability in developed countries than any other group of illnesses, including cancer and heart disease [4]. The waiting lists to receive adequate mental care are constantly growing [5], and costs associated with mental disorders are considerable and rising (e.g. [6]). As an example, financial costs associated with mental disorders in the Netherlands are 4 billion euro annually, [7] not to mention the degree of human suffering these disorders induce in patients and their families [8]. Studies show that the onset of most mental disorders commonly fall within the timeframe from early teens to mid-twenties [9, 10], negatively affecting quality of life [11], life expectancy [12] social functioning, ability to work [11], and (self-)stigmatization [11, 13, 14]. Even though a proportion of mental disorders in young people resolves without professional help in their late twenties, much pain, unrealised potential, disability, or premature death will have occurred by then. Physiological changes that occur during puberty strongly affect behaviour and emotional functioning, creating a disjunction between physical, intellectual, and psychosocial maturity [15]. Adolescence and early adulthood is also a time of major structural and functional changes in the brain, driven by a series of maturational processes that result in the refinement of the neuronal circuitry and a recalibration of the inhibitory-excitatory balance, particularly in the frontal cortex [16]. There is an urgency to reform our current mental health care system to address the needs of youth. Young people who are experts by experience indicated the following problems with current Dutch system of mental health care: 1) The focus is too much on diagnosing and treatment protocols while young people want to receive individualized help for their problem, 2) When young people receive a psychiatric diagnosis, treatment protocols are focused on that particular diagnosis while other problems are often left untreated (e.g. drug use, low self-esteem), 3) Many psychiatric services are not appealing to youth because people of all ages are treated there and the approach is not aligned with the needs of young people, 4) The threshold to seek professional help is high because of fear of stigma associated with mental illness, 5) The gap between youth and adult mental health services at age 18 hampers continuity of care [17, 18].

Prevention and early intervention are essential for reducing the psychosocial and economic impact of any potentially serious health condition [19, 20]. The preventive approach has worked well in many other fields of medicine such as oncology and cardiology, vastly improving prognosis (e.g. [21, 22]). However, in the Dutch system of mental health care, people need to get a DSM diagnosis to receive reimbursement for their healthcare costs, even though at the stage of a DSM diagnosis, a mental disorder has worsened and prognosis has deteriorated [23]. Intervention in an earlier phase of possible onset of mental disorders is more effective than intervention after onset of a full-blown disorder, because psychosocial and neurobiological damage is less extensive and subjects are more responsive to treatment at this stage [19, 20, 24]. Due to the effectiveness of prevention and early intervention, clinical staging in psychiatry is receiving more attention worldwide [25, 26]. The model provides clear cut-offs between stages, making it usable in clinical practice [27]. In the current project, we focus on youth in stages 1a: Help-seeking individuals with mild symptoms and mild functional impairment, and stage 1b: People with attenuated syndromes with partial specificity, often with mixed or ambiguous symptoms and moderate functional impairment. Inter-rater reliability of this model is good, with 90% concordance between experienced raters. See Table 1 for the full overview of the clinical stages.

**Table 1:** Clinical staging model (Adapted from Hickie and colleagues [25])

Stage	Description
Stage 0	Asymptomatic individuals at risk of a disorder who have not yet presented for care
**Stage 1a**	**Help-seeking individuals with mild symptoms and mild functional impairment**
**Stage 1b**	**People with attenuated syndromes with partial specificity, often with mixed or ambiguous symptoms and moderate functional impairment**
Stage 2	People with discrete disorders: clear episodes of psychotic, manic, or severe depressive symptoms
Stage 3	People with recurrent or persistent disorders
Stage 4	People with severe, treatment-resistant, or unremitting disorder

Early detection and treatment of mental health complaints as well as personalized health care for young people have been successfully implemented in Australia guided by the leadership of Patrick McGorry with over 120 Headspace centres that aim to prevent chronicity and referral to specialized mental health services [28, 29]. Young people aged 12–25, and their family and friends can seek help for general health and education problems, drug use and (emerging) mental health complaints. Evidence-based interventions are provided in a stepped-care manner, i.e. the least invasive and harmless evidence-based interventions are employed first [15, 30, 31]. The Headspace model has bridged the traditional service gap between youth and adult mental health services by not cutting off access to services at age 18 [32].

However, several studies show only a third of young people seek help for their mental health problems [33, 34], and most individuals receive mental health care at a much later stage [35–37]. Since the step to seek help remains relatively large, within a national youth e-mental health service (eHeadspace Generation Next), Australia successfully implemented the Moderated Online Social Therapy (MOST+); an evidence based digital interactive peer and clinical moderated treatment platform for youth with beginning mental health problems [38]. Inspired by the Australian Headspace approach, Headspace centres have been implemented in e.g. the Netherlands, Denmark and Ireland. However, the MOST+ platform has not been implemented yet in Europe. This project aims to support young people (between the ages of 16–25; and possibly at a later stage between the age of 12 and 25) with beginning mental health problems by translating, adjusting and implementing the MOST+ digital platform to the situation in the Netherlands in collaboration with a youth panel. For the Dutch version of the platform, we use the name ENgage YOung people earlY (ENYOY). Research shows that the MOST+ site leads to minimal drop out and enhances continuation of treatment effects [32, 38]. This model has the potential to realise the goal of specialized treatment by providing cost-effective, non-stigmatising, constantly available support to young people suffering from mental health problems.

Furthermore, the relation between stress and mental disorders, such as depression, has been subject of research [39] and less stress reactivity has been shown to predict symptom improvement in children with anxiety disorders [40]. Different patterns in parasympathetic and sympathetic nervous system activation respond to different kinds of treatments. Thus, stress biomarkers could be useful for individualized prognosis and treatment selection [41]. Developments in ambulatory sensor and information technology enable researchers to equip part of the young people who are on the platform with a smart-watch for minimally-invasive stress monitoring, aiming to clarify the relation between stress and their activities, and enhancing their autonomy, insight and resilience.

## Objectives

With this implementation study, we aim to reduce the chance that young people develop a serious mental disorder, escalation of problems with resulting chronicity and long treatment trajectories in the Netherlands. With a stepped care and positive psychology approach we strengthen the natural recovery potential of young people with emerging mental health complaints [8, 32]. We will use the following parameters to investigate the effects and functionality of the ENYOY platform in the Netherlands: 1) empowerment, psychosocial functioning, quality of life, hope and recovery, 2) mental symptoms of young people 3) stress biomarkers 4) user experiences. We will employ the formulation of health as “the ability to adapt and to self-manage” [42]. In Australia over one-third of MOST+ clients had significant improvements in psychological distress (K10 [43]) and a similar proportion in psychosocial functioning (SOFAS [44]). Sixty per cent of clients showed significant improvement on one or both measures [31]. Our hypothesis is that the project will attain similar results of improvement among young people in psychological distress and psychosocial functioning with ENYOY platform in the Netherlands. Additionally, stress is one of the most-studied pathways to mental disorders. Stress indicators may prove useful as biomarkers of psychopathology. This project will contribute to determining the utility of such biomarkers for prognosis and the development of new treatment options. We will assess stress parameters with a smart-watch and time series analysis [45, 46]. A significant reduction in stress parameters is expected at follow up. Another important objective in this project is to engage young people themselves to share their concerns, ideas and suggestions with the research team by enhancing participation of youth experts who experienced mental health problems and enable the project team to learn from their experience.

Objectives:

1. To investigate the effects of the adapted MOST+ platform in the Netherlands on empowerment, psychosocial functioning, quality of life, hope and recovery as well as mental symptoms of young people.
2. To investigate the utility of biomarkers for prognosis and the development of new treatment options.
3. To engage young people themselves to share their concerns, ideas and suggestions with the research team by enhancing participation of youth experts who experienced mental health problems and enable the project team to learn from their experience.
4. Analyze feedback of young people on the platform concerning usability, user friendliness, accessibility, acceptability, connection among peers, and contribution of the platform to the lessening of mental health complaints and increase of positive mental health.

## Trial design

This study has a longitudinal cohort mixed-method design (qualitative and quantitative research) and equivalence framework. It has a single group, therefore allocation-ratio is not applicable.

## Methods: participants, interventions and outcomes

### Study setting

The study setting is a digital treatment environment (ENYOY). The Dutch participants will be recruited via Dutch the @ease centers in Amsterdam and Maastricht, plus via social media. The physical study site is the Amsterdam UMC, though most of the interviews with participants, measures, and conversation and measurements will take place online, partly because of the current COVID-19 pandemic.

### Eligibility criteria

#### Inclusion criteria

- Age 16–25. The Amsterdam University Medical centres (IRB) requested to begin the study with young people aged 16–25 instead of 12–25. Possibly at a later stage in the study, after positive first results, the age-group 12–25 can be included.

- Help-seeking for mental health concerns in stages 1a or 1b.

- Being able and willing to consent.

#### Exclusion criteria

- Mental disorder in clinical stages 2–4.

- Acute risk of self-harm requiring urgent intervention (i.e. suicidal ideation with a current plan and intent to enact this plan).

The interventions will be supported by expert mental health and peer moderators to ensure the safety of the intervention and to directly support participants with moderation, chats and referral to other forms mental health care if needed.

### Who will take informed consent?

Once a young person shows interest in the study, the participant information and informed consent forms will be sent. The project staff will inform if this information is received, and after one week the young person will be asked if he/she has any questions, and wants to participate. If a young person wants to participate, an intake will take place to determine if the ENYOY platform would be helpful for the young person, or if other care is needed. If there is a match, within one week a research assistant from the project staff will obtain informed consent before the baseline measurements take place. When at a later stage inclusion of young people aged 12–25 is possible; a parent or guardian as well as the young person will sign informed consent.

### Intervention description

The Australian MOST+ platform is based on evidence-based modules for mental health complaints, e.g. Cognitive Behavioral Therapy and mindfulness. Each participant will start on the platform with the ‘onboarding’ process. This is a process where clinical presenting issues and character strengths of the individual will be measured (based on Seligman’s positive psychology framework [47, 48] aligned with the empirically supported VIA questionnaire [49, 50]), following which the algorithm of the platform makes an individual tailored treatment journey suggestion for that person. The project group will consider the following modules (building blocks) of the MOST+ platform for translation and adaptation in the Netherlands:

1. Guided therapy journeys: A program of engaging evidence-based therapy (Mindfulness-Based Cognitive Therapy, Cognitive Behavioral Therapy, Meta-Cognitive Therapy, and Social-Cognition strategies) tailored to young person’s needs by automated processes and ENYOY therapists. For example, programs for social anxiety challenges, worrying or depressive symptoms where themed content can be curated to needs of individual. Transdiagnostic processes are found in the domains of attention, memory/imagery, thinking, reasoning and behavior. Activities within a therapy journey entail:
  - Reflective Actions: Behavioral or cognitive experiments to improve the ability to notice thought processes in order to build insight and self-awareness of these processes.
  - Regular Actions: Assist the young person trial learnings in real-world contexts, hereby generalizing adaptive coping strategies and behaviors (increasing self-efficacy and challenging cognitions via experiments).
  - Therapy Comics: Comics that offer an engaging, powerful and accessible means of understanding mental ill health challenges. Narratives offer young people new ways of negotiating the challenges of mental illness.
  - Talking Points: Subjects that provide young people with an opportunity to share their effective coping strategies. This encourages social problem solving and peer modeling/learning.
  - Strength-Based Actions: Actions to assist the young person to use their innate character strengths to overcome mental health challenges. Helps young person utilize their strengths (rather than emphasis on alleviating symptoms).
  - Myth Busters: Information to help challenge internal stigma and help normalize and validate young people’s experiences.
  - Inspirational quotes.
  - Fun Facts: Facts to provide psychoeducation on a particular topic, which can also assist with de-stigmatizing particular experiences.
2. Personalized therapy toolkit: A personal library of young person’s therapy work and favorite strategies. Therapy activities that are helpful are automatically saved to the toolkit, and young people can save content in their toolkit.
3. Safe online social network: A moderated virtual support network of other young people with a shared experience of mental health problems, there to support young persons if and when they need it on their recovery journey
4. Professional online support: A base wrap-around support from online peer workers and ENYOY therapists.

In summary the ENYOY platform will contain:

i) peer-to-peer online social networking;
ii) individually tailored interactive psychosocial interventions;
iii) involvement of expert mental health and peer moderators to ensure the safety of the intervention and to directly support participants with moderation, chats, and referrals to other forms of health care if needed.

Concerning clinical safety, the MOST+ safety protocol builds on the excellent track record of the Australian group managing 24/7 available digital social media interventions safely [51]. Manual and automated procedures will be followed. Firstly, information related to clinical risk (posts or messages) will be screened twice daily by therapists. Secondly, MOST+ incorporates an automatic alert system which detects information consistent with increased risk of suicide (via automated monitoring of self-harm-related terms posted in the social feed using previously validated approaches [51]. Any detected increased risk will activate the safety protocol. The therapist will conduct a telephone risk assessment and, where necessary, implement one or more of the following procedures: i) inform the treating clinician (if relevant); ii) inform nominated emergency contact; iii) liaise with suitable emergency services. Additional safety features include a reporting function for users and visible 24/7 emergency numbers. This safety protocol has been approved by three ethics committees and successfully implemented in four pilot studies and two ongoing RCTs [52]. Concerning ICT safety, the platform has been adjusted to European laws and safety standards together with a privacy officer.

### Criteria for discontinuing or modifying allocated interventions

If mental health complaints of a participant worsen, e.g. develop into stage 2 or above (see introduction), together with the participant the assigned clinical moderator and ENYOY team will look into finding more intensive care. This does not exclude them per se from the platform and current study; they can have additional help and still participate. The form and intensity of additional care will be logged. Following discontinuation, treatment will be offered on a “need for care-clinician’s choice” basis according to existing clinical practice guidelines. Wherever possible, subjects who discontinue the study prior to 12 months from entry will be followed with full regular assessments as per the subjects who continue with the protocol treatment.

### Strategies to improve adherence to interventions

The adherence to the platform is continually measured by the platform itself. The clinical moderators have access to usage data as well as the percentage of the personal pathway completed. With the weekly check-up they have with each participant, this will be discussed. In weekly supervision sessions among clinical-, peer moderators, and supervisors adherence of participants will be discussed, and thought about ways to improve when found low.

### Relevant concomitant care permitted or prohibited during the trial

All other forms of care, co-interventions, are permitted during the trial. Co-interventions will be registered with the treatment document sheet, and could be used for explorative analysis.

### Outcomes

Data will be collected digitally using CASTOR at baseline and after 3, 6 and 12 months.

Outcome measures:

- Psychological Distress assessed with the Kessler Psychological Distress Scale (K10, [43]).
- Social and Occupational Functioning Assessment Scale (SOFAS, [44]).
- The positive health instrument assesses empowerment, psychosocial functioning, quality of life, hope and recovery as well as mental symptoms [42, 53, 54].
- Stress biomarkers with a smart-watch. Participating clients who are interested will receive a smart-watch that continuously monitors relevant physiological markers of stress during 3 weeks. Available technology (e.g. the TIQ watch E2) is validated, and can measure heart rate variability. In this way, both incidental and cumulative stress can be validly assessed in a naturalistic setting. The participants will receive a short introduction for use about the watch. They can check their stress under different circumstances to discover the relationship between their activities and stress. The watch could also be used during their visits to the ENYOY site to measure the changes in stress level during exercises on the platform, such as mindfulness exercises, self-compassion elements and the anxiety program.
- “Websites journeys” of visitors (number of visits, number of selected activities etc.) will be collected. The website journeys will be anonymously logged under a system generated ID number and session number.
- Open questions asked in an digital online meeting (using MS teams) regarding usability, user friendliness, accessibility, acceptability, connection among peers, and contribution of the platform. The focus will lie on subjective meaning-giving and context, thereby focusing on the individual. The experience and interpretation of the participants will be explored. The presented results will form descriptions and insights into the core constructs, and help improve the ENYOY platform further.

### Participant timeline

**Fig. 1:**
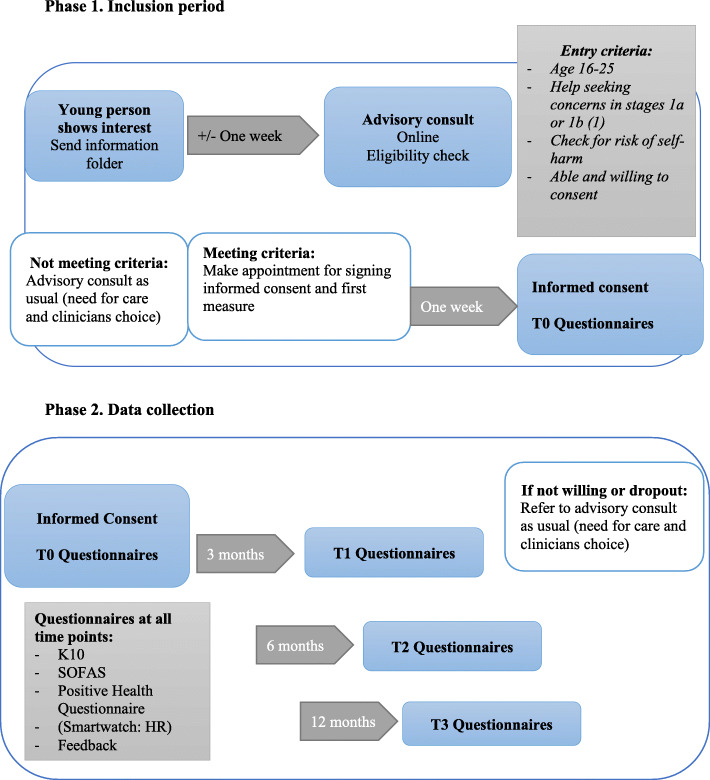
Participant timeline

For the participant timeline, see Fig. 1. During the 12 months, participants can use the platform as much as needed.

### Sample size

Rickwood et al. [31] reported significant change (effect size Cohen’s d > .5) in the K10 and SOFAS between their first and last Headspace service ratings. In Australia over one-third of Headspace clients had significant improvements in psychological distress (K10 [43]) and a similar proportion in psychosocial functioning (SOFAS [44]). Sixty percent of clients showed significant improvement on one or both measures. These outcomes were derived from face-to-face services, however similar effects are suspected since a similar framework will be used. Using a paired samples t-test with 80% power and an alpha of 0.01, we would need a sample of 43 subjects to find similar results. Drop-out rates for digital treatment interventions for young people with beginning mental health complaints have been found to range between 2 and 73% [55]. Inclusion of 125 subjects in the study would give us ample power to investigate our main hypothesis.

### Recruitment

Due to the COVID-19 crisis, there are now too few face-to-face appointments and intakes in the Headspace and other participating centers. Therefore, young people will be recruited via social media and websites for young people as well. Young people aged 16–25 years (and possibly later 12–25 years) who show interest will receive an online intake via a secured environment (MS Teams). They will be assessed for the in- and exclusion criteria and demographic variables will be collected. When eligible, a second appointment will take place, where the informed consent can be signed, the first measurements take place (see flow diagrams above), and an account for the platform will be set up.

## Data collection and management

### Plans for assessment and collection of outcomes

To determine eligibility, a clinical interview and screening will take place. In intervision with a team of psychologist the operationalization of the clinical staging model [25] will be used to determine the stage of mental health problems.

The data on T0, T1, T2 and T3 will be collected in CASTOR, which enables safety and anonymous storing of outcomes. The research team will be trained in how to collect the measures (questionnaires) and in how to use CASTOR.

The K10 is an instrument measuring psychological distress using 10 questions. Strong psychometric qualities have been found [43].

The Social and Occupational Functioning Scale (SOFAS) rates the social and occupational functioning of individuals. It is different from the Global Assessment of Functioning (GAF) known from the DSM-IV since it does not take into account psychological symptoms, and does take into account impairments due to a general medical condition. It is usually used for the evaluation of the current time period [44]. The SOFAS has a good reliability and validity [56].

The Positive Health Instrument is an instrument that assesses empowerment, psychosocial functioning, quality of life, hope and recovery as well as mental symptoms [54]. The instrument is relatively new and still being researched [53]. Overall good reliability was found; the validity has not been researched yet [57].

### Plans to promote participant retention and complete follow-up

Participants will receive financial compensation for their time (9 euros per hour) and travel costs will be fully reimbursed at the end and completion of all measurements. All deviations form protocol will be reported.

### Data management

Upon enrolment, participants will receive a participant number. In a document saved with a password on a protected computer environment the key will be saved. All data will be stored using solely the participant number. As mentioned before, CASTOR will be used for safe storage. All research assistants will be trained to promote safety, and data quality. Random check-ups by other research assistants will be done to assure data is saved correctly. Moreover, data will always be processed by two research assistants, to promote accurateness.

### Confidentiality

As stated above, the personal information of participants will be stored separately from the data of the research, and is only accessible for the selected research staff, with a password. Moreover, potential participants will get a screening number, using the same process of separate data form personal information. If a young person is interested in participation, he/she can send an e-mail to a protected mailbox, thereby ensuring safety also in the shown interest. Only the research team has access to the mailbox. After the trial the data will be deidentified and saved for 15 years, in the controlled environment.

## Statistical methods

### Statistical methods for primary and secondary outcomes

The study has a longitudinal cohort design. Following Rickwood et al. [31] frequencies of each primary presenting concern will be calculated, and age group and sex differences will be assessed by X2 analyses with Bonferroni correction for multiple comparisons. Changes in social functioning (SOFAS) and psychological stress (K10) measures over time will be analyzed in two ways [58]. First, repeated measures multivariance analysis of covariance (MANCOVA) will be used to assess aggregate changes over time in K10 and SOFAS scores according to time point. Time on the platform, therapy pathway completed, number of visits to the ENYOY platform, age group and/or sex will be used as covariates. The statistical relationship between K10 and SOFAS scores will be expressed as a Pearson product-moment correlation coefficient. Differences between the characteristics of clients who provide follow-up data and those who did not will be analyzed by logistic regression. Second, significant change, reliable change and clinically significant change scores will be calculated for the K10 and SOFAS data, as increasingly conditional indicators of change. The criterion for significant change is a moderate effect size (0.5) or greater for the degree of change. The reliable change index (RCI) (indicating reliable improvement or decline) and clinically significant change index (CSI) (cut-off point at which the person is more likely to belong to a non-clinical rather than a clinical population) will be determined using Jacobson and Truax’s method [59]. For the K10 scores, the RCI will be estimated as a 7-point change using reliability coefficients reported for a normative group (age group, 16–24 years) in the 2007 National Survey of Mental Health and Wellbeing [60], and will calculated individually on the sample. Using the same norms, the CSI cut-off is estimated to 23 points. For the SOFAS data, an RCI score of 10 will be used [31]. Positive health and stress biomarkers outcome measures will be analyzed using regression analysis and an MANCOVA to investigate relationships between stress biomarkers, symptoms, empowerment, hope, quality of life and recovery longitudinally. Time on the platform, therapy pathway completed, number of visits to the ENYOY platform, age group and/or sex will be used as covariates. Explorative analyses can be used to research the effect of additional received treatments. Finally, 10 participants will be asked additional questions concerning usability, user friendliness, accessibility, acceptability, connection among peers, and contribution of the platform. The transcripts firstly will be coded using open methods by labeling and merging synonyms, this to ensure data reduction. Secondly the labels will be categorized by axial coding, thereby resulting in systematic arrangement. Finally, selective coding will be done to couple the research question to the central constructs.

### Interim analyses

An interim analysis will be conducted to investigate safety and usefulness in participants aged 16–25. If so, inclusion of those aged 12–15 will be considered. In accordance to section 10, subsection 4, of the WMO, the sponsor will suspend the study if there is sufficient ground that continuation of the study will jeopardise subject health or safety. The sponsor will notify the accredit METC without undue delay of a temporary halt including the reason for such an action. The study will be suspended pending a further positive decision by the accredited METC. The investigator will take care that all subjects are kept informed.

### Methods in analysis to handle protocol non-adherence and any statistical methods to handle missing data

Multiple imputation (MI) will be used to handle missing data.

### Plans to give access to the full protocol, participant level-data and statistical code

Participant-level dataset and statistical code can only be shared upon official request with METC permission. The study protocol will be published and will thereby be accessible.

## Oversight and monitoring

### Adverse event reporting and harms

The safety protocol will be consulted in the case of serious adverse events and adverse events. All serious adverse events and adverse events will be categorized, recorded and reported to sponsor, local trial site, clinical trials unit (CTU), and trial oversight committees. The investigator will report all SAEs to the sponsor without undue delay after obtaining knowledge of the events. The sponsor will report the SAEs through the web portal ToetsingOnline to the accredited METC that approved the protocol, within 15 days after the sponsor has first knowledge of the serious adverse reactions.

All AEs will be followed until they have abated, or until a stable situation has been reached. Depending on the event, follow up may require additional tests or medical procedures as indicated, and/or referral to the general physician or a medical specialist.

SAEs need to be reported till end of study within the Netherlands, as defined in the protocol.

### Plans for communicating important protocol amendments to relevant parties (e.g. trial participants, ethical committees)

Amendments are changes made to the research after a favorable opinion by the accredited METC has been given. All amendments will be notified to the METC that gave a favorable opinion. All parties (e.g. investigators, participants, researchers, financers) will receive updates on changes.

## Dissemination plans

The results of scientific research involving human subjects must be disclosed unreservedly and there are no objections on this regard. The participants can request to receive the results of the study. Moreover, presentations for the general public, and other healthcare professionals will take place in order to make found results accessible.

## Discussion

This trial will specifically address young people with emerging mental health complaints, and offer a new approach for preventive intervention in the Netherlands. Treating emerging mental health problems before they develop into more chronic mental illnesses, could contribute to decreasing the burden of mental health problems for youth, and decreasing mental health associated costs [6–8]. Considering the waiting lists in (child and adolescent) psychiatry and the increase in suicides among youths, early low-threshold and non-stigmatizing help for young people with emerging mental health symptoms is of crucial importance. The transition from child to adult psychiatry holds a risk for disruption in continuity of care [61, 62]. This project aims to bridge the gap between child and adolescent and adult psychiatry. Possible limitations of the study are, that there is a chance that only individuals that actively search for help will participate in the study, thereby not reaching a diverse sample of young people who need help. To overcome this limitation, we attempt to involve several parties in the recruitment, such as universities and general practitioners. Still, certain young people that stay under the radar may not be reached. This would limit the generalizability of our results regarding the effectiveness of the platform to the youth population. Moreover, this study is not an RCT, meaning the level of evidence is lower. Further, the longitudinal research design has the risk of participants dropping out over time. However, since the effectiveness of the platform has extensively been researched in Australia for over ten years, and the focus of this research is on implementation of the platform, we believe the chosen design matches the research questions best. This trial will contribute to the implementation of a transdiagnostic, digital, clinical – and peer- moderated, indicative prevention treatment platform for youth with beginning mental health complaints in the Netherlands. Moreover, this is the first time the platform is translated and adjusted to a country outside of Australia. The project could contribute to the implementation of the platform in other countries as well.

## Data Availability

The data resulting from the current study will not be publicly available. The data, protocol, statistical code and a list of study sites are available from the corresponding author on reasonable request.

## Abbreviations

ANBI: Algemeen Nut Beogende Instelling (english: Public Benefit Institution)
ABR: ABR form, General Assessment and Registration form, is the application form that is required for submission to the accredited Ethics Committee (In Dutch, ABR = Algemene Beoordeling en Registratie)
AE: Adverse Event
AR: Adverse Reaction
CA: Competent Authority
CCMO: Central Committee on Research Involving Human Subjects; in Dutch: Centrale Commissie Mensgebonden Onderzoek
CV: Curriculum Vitae
DSMB: Data Safety Monitoring Board
ENYOY: ENgage YOung people earlY
EU: European Union
EudraCT: European drug regulatory affairs Clinical Trials
GCP: Good Clinical Practice
IB: Investigator’s Brochure
IC: Informed Consent
IMP: Investigational Medicinal Product
IMPD: Investigational Medicinal Product Dossier
METC: Medical research ethics committee (MREC); in Dutch: medisch ethische toetsing commissie (METC)
(S)AE: (Serious) Adverse Event
SPC: Summary of Product Characteristics (in Dutch: officiële productinfomatie IB1-tekst)
Sponsor: The sponsor is the party that commissions the organisation or performance of the research, for example a pharmaceutical company, academic hospital, scientific organisation or investigator. A party that provides funding for a study but does not commission it is not regarded as the sponsor, but referred to as a subsidising party.
SUSAR: Suspected Unexpected Serious Adverse Reaction
Wbp: Personal Data Protection Act (in Dutch: Wet Bescherming Persoonsgevens)
WMO: Medical Research Involving Human Subjects Act (in Dutch: Wet Medisch-wetenschappelijk Onderzoek met Mensen
